# Nonlinear Relationship Between Macrocytic Anemia and Decompensated Hepatitis B Virus Associated Cirrhosis: A Population-Based Cross-Sectional Study

**DOI:** 10.3389/fphar.2021.755625

**Published:** 2021-09-20

**Authors:** Tian-Yu Zhao, Qing-Wei Cong, Fang Liu, Li-Ying Yao, Ying Zhu

**Affiliations:** Liver Disease Center of Integrated Traditional Chinese and Western Medicine, The First Affiliated Hospital of Dalian Medical University, Dalian, China

**Keywords:** macrocytic anemia, decompensated HBV associated cirrhosis, MELD score, degree of liver damage, mean corpuscular volume

## Abstract

**Background:** Mean corpuscular volume (MCV) is major used as an indicator for the differential diagnosis of anemia. Macrocytic anemia in decompensated cirrhosis is common. However, the relationship between macrocytic anemia and decompensated hepatitis B virus (HBV) associated cirrhosis has not been fully addressed.

**Methods:** In this cross-sectional study, a total of 457 patients diagnosed decompensated HBV associated cirrhosis who met all inclusion criteria from 2011 to 2018 were analyzed. Association between macrocytic anemia and the liver damaged (Model for End Stage Liver Disease (MELD) score) were examined using multiple logistic regression analyses and identified using smooth curve fitting.

**Results:** Compared with normocytic anemia, MCV and MELD are significantly positively correlated in macrocytic anemia (*p* < 0.001). A non-linear relationship of MCV and MELD association was found though the piecewise linear spline models in patients with decompensated HBV associated cirrhosis. MCV positive correlated with MELD when the MCV was greater than 98.2 fl (regression coefficient = 0.008, 95% CI 0.1, 0.4).

**Conclusion:** Macrocytic anemia may be a reliable predictor for mortality because it is closely related to the degree of liver damage in patients with decompensated HBV associated cirrhosis.

## Introduction

Liver cirrhosis is a frequent end stage of liver disease, which itself results from a long-term process of fibrosis and sustained inflammation and leads to chronic liver disease (Schuppan and Afdhal, 2008). Hepatitis B virus (HBV) infection remains a very common liver disease (Nguyen et al., 2020), over 70% of infected cases are diagnosed as liver cirrhosis in China (Xiao et al., 2019). During the natural course of the disease, cirrhosis has transitioned from the compensation stage to the decompensation stage, through the developmental processes of one of the following serious complications: variceal hemorrhage, spontaneous bacterial peritonitis (SBP), encephalopathy, or jaundice. The 5-years liver related decompensated incidences in patients with compensated cirrhosis are 15–20%, 5-years survival rate for patients with compensated cirrhosis is approximately 84%, while for patients with decompensated cirrhosis, survival rate drops to 14–35% (Peng et al., 2012).

Anemia is now identified as an important predictor of adverse outcomes in liver cirrhosis patients, such as the development of acute-on-chronic liver failure (ACLF) in outpatients with cirrhosis and hepatocellular carcinoma mortality rates (Finkelmeier et al., 2014; Piano et al., 2017). Mean corpuscular volume (MCV) is defined as a measure of the average volume of a red blood cell, anemia is classified into three categories depending on the level of the patient’s MCV: macrocytic anemia (>100 fl), normocytic anemia (80–100 fl) and microcytic anemia (<80 fl). A study has indicated that an increase MCV level was correlated with the prognosis of liver cancer (Yoon et al., 2016). However, the exact mechanisms behind the relationship between MCV and liver function damage degree in patients with decompensated hepatitis B virus-related cirrhosis is still unknown. The model for End Stage Liver Disease (MELD) score was a preferred tool to use to predict the short-term mortality of end-stage liver disease and measure cirrhosis severity (Kamath and Kim, 2007). It had been considered to be an important predictor of survival for end-stage liver disease caused by many etiologies and was considered an organ allocation strategy for liver transplantation more accurate than Child-Pugh score since its application in the United States in 2002 (Wiesner et al., 2003; Bambha et al., 2004). Analysis demonstrated that the greater MELD scores (≥15), the greater risk of death from liver disease, as well as showed a significant survival benefit from liver transplantation compared to lower MELD scores (<15) (Merion et al., 2005). Thus, higher MELD score are expected to indicate worse liver function.

Previous a study showed that the relationship between MCV and MELD (Yang et al., 2018), however, this relationship has not been well studied. Therefore, we investigated whether MCV is independently associated with MELD in HBV-associated decompensated cirrhosis.

## Methods

### Characteristics of the Participants

**FIGURE 1:**
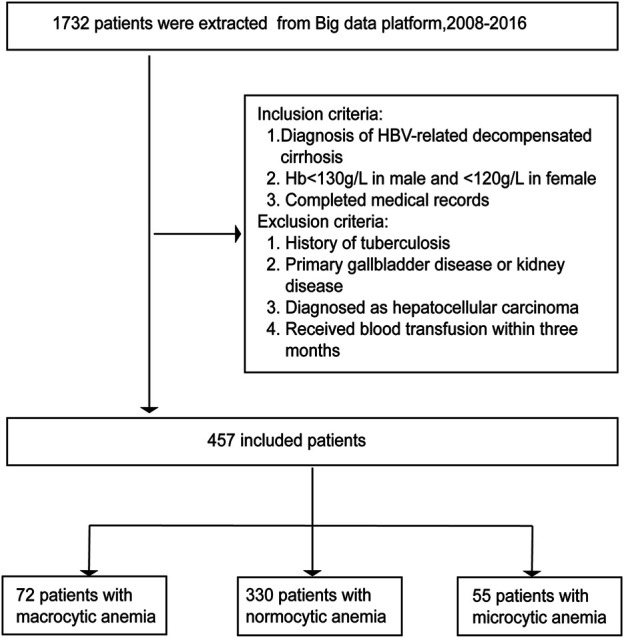
Flow chart for the selection of patients.

This is a retrospective study from the Big Data Platform of the First affiliated hospital of Dalian Medical university from May 2011 to April 2018, our data consists of 1732 patients with decompensated HBV associated decompensated cirrhosis. Our research used the International Classification of Diseases codes to identify decompensation cirrhosis with HBV hospitalized patients. Decompensated Cirrhosis in Patients with hepatitis B according to the China’s Guidelines for the Prevention and Treatment of Chronic Hepatitis B (The guidelines of prevention and treatment for chronic hepatitis B (2019 version), 2019), 1) HBsAg carrier for study population≥6 months; 2) confirm the presence of cirrhosis according to biochemical, radiological, endoscopic and histological criteria; 3) at least one episode of ascites, spontaneous bacterial peritonitis, hepatic encephalopathy, or variceal bleeding (Jang et al., 2015). The WHO defined anemia as a hemoglobin level <130 g/L in male and <120 g/L in female (McLean et al., 2009). Two investigators reviewed the charts of all patients, Any discrepancies between the two investigators will be adjudicated by a senior physician. 457 patients who met all inclusion criteria and none of the exclusion criteria were enrolled into the study. This cross-section hospital-based, observational study was conducted in a University Hospital (Figure 1).

The research protocol was reviewed and approved with a waiver of written informed consent by the Ethics Committee of the First affiliated hospital of Dalian Medical university, informed consent by telephone was obtained from each participant. All the methods were performed in accordance with relevant guidelines and regulations.

### Data Collection

Demographic characteristics were obtained from face-to-face communication with patients or their families when the patient was admitted to our hospital. Blood samples were taken from the patients on an empty stomach for more than 10 h after the whole night and fast sent to the laboratory assessments. Having more than one cigarette per day is considered as smoking and alcohol intaking more than 20 g per day for at least a year is considered as drinking (Kim et al., 2013; Carter et al., 2015). Estimated GFR (eGFR) formula was derived from the modification of diet in renal disease (MDRD). Regrettably, due to missing data, HBV DNA data and body mass index (BMI) were excluded from this study (K/DOQI clinical practice guidelines for chronic kidney disease: evaluation, classification, and stratification, 2002; Ma et al., 2006).

### MELD Score

Using the following formula to calculate the MELD score: 9.57 × loge (creatinine mg/dl) + 3.78 × loge (bilirubin mg/dl) + 11.2 × loge (INR) + 6.43, where INR is the international normalized ratio and 6.43 is the constant of the etiology of liver disease (Kamath and Kim, 2007).

### Statistical Analysis

Categorical variables were described in counts (percentages) and continuous variables as means ± standard deviation (SD). Patients were distributed into 3 groups by mean corpuscular volume (MCV) classification. The variables were followed normal distribution and homogeneous in variance. The levels within 3 groups of the continuous variables were analyzed using one-way ANOVA. categorical variables were analyzed using Chi-square test. To evaluate the relationship between the MELD score and macrocytic anemia were analyzed using univariate and multivariate linear regression analyses. Only variables with a *p*-value < 0.05 in the univariate analyses were planned to be included in the multivariate model. The possible linear and nonlinear models were used to assess the relationship between MELD and MCV by multiple linear regression models and two-piece piecewise regression models adjusted for sex, age, smoking, drinking, SBP, DBP. We then performed stratified analyses in order to further explore potential modifier on the MELD-MCV association.

All data analysis and form generation were produced using the statistical package R (http://www.R-project.org, The R Foundation) and Empower (R) (www.empower stats.com; X&Y Solutions, Inc. Boston, MA). The results data were considered statistically significant When *p*-value was <0.05.

## Results

### Characteristics of the Participants

The baseline characteristics of subjects with anemia were divided into three groups (Table 1). Among the 457 participants in this analysis, 330/457 patients (72.2%) of the anemic cases had normocytic anemia, with the remaining 127 (27.8%) having macrocytic (*n* = 72) and microcytic anemia (*n* = 55). The cohort was 74.2% male, had a mean age of 65.5 (SD = 12.9). In addition, we found significantly higher expression levels of serum bilirubin, international normalized ratio (INR) and MELD score in macrocytic anemia when compared to normocytic or microcytic anemia. However, no significant differences were found in age, gender, smoking, drinking, diabetes, hypertension, systolic blood pressure, diastolic blood pressure, creatinine, eGFR, albumin, alanine aminotransferase (ALT), aspartate aminotransferase (AST), alkaline phosphatase (ALP), γ-glutamyltranspeptidase (γ-GT).

**TABLE 1:** Baseline Characteristics of participants (*N* = 457).

Variable	Macrocytic anemia	Normocytic anemia	Microcytic anemia	*p*-value
No. of participants	72	330	55	
Mean corpuscular volume, fl	105.40 ± 4.49	91.14 ± 5.09	72.92 ± 5.16	<0.001
Age, years	66.29 ± 13.79	65.32 ± 12.65	65.78 ± 13.72	0.617
Sex				0.120
Male, n (%)	60 (83.33)	237 (71.82)	42 (76.36)	
Female, n (%)	12 (16.67)	93 (28.18)	13 (23.64)	
Smoke, n (%)	28 (41.18)	98 (31.11)	23 (43.40)	0.091
Alcohol, n (%)	25 (37.88)	88 (27.76)	19 (35.85)	0.171
Diabetes, n (%)	8 (11.11)	58 (17.58)	11 (20.00)	0.332
Hypertension, n (%)	9 (12.50)	55 (16.67)	12 (21.82)	0.376
Hemoglobin, g/L	102.48 ± 21.07	102.22 ± 18.98	73.15 ± 18.14	<0.001
Hemoglobin, categorical recoded, n (%)				<0.001
>90	57 (79.17)	245 (74.24)	9 (16.36)	
60–90	11 (15.28)	78 (23.64)	36 (65.45)	
<60	4 (5.56)	7 (2.12)	10 (18.18)	
Blood glucose, mmol/L	5.58 ± 2.00	6.19 ± 3.22	6.21 ± 2.57	0.588
SBP, mmHg	128.36 ± 17.88	128.86 ± 19.29	127.64 ± 19.71	0.715
DBP, mmHg	76.41 ± 10.40	77.56 ± 11.88	79.62 ± 12.71	0.378
Bilirubin,μmol/L	56.3 (25.6–120.2)	40.4 (25.1–75.0)	34.1 (19.2–71.6)	<0.001
Creatinine,μmol/L	63.5 (50.5–93.2)	62.0 (49.0–86.0)	66.0 (55.5–90.5)	0.209
INR	1.50 ± 0.69	1.27 ± 0.27	1.30 ± 0.22	<0.001
eGFR				
mL/min/1.73 m^2^	110.0 (74.2–761.2)	112.3 (75.4–2,362.1)	121.5 (83.0–377.2)	0.091
ALB	31.31 ± 6.82	31.04 ± 6.06	33.04 ± 5.87	0.090
AST	60.5 (33.2–117.8)	59.0 (34.0–105.8)	47.0 (31.0–93.5)	0.518
ALT	37.5 (23.0–88.0)	42.0 (24.0–74.0)	37.0 (23.0–67.0)	0.864
ALP	121.0 (81.8–204.5)	123.5 (89.0–186.8)	138.0 (81.5–244.0)	0.924
GGT	80.5 (36.8–190.5)	94.0 (45.0–217.5)	107.0 (43.5–364.5)	0.663
MELD	17.02 ± 6.94	14.82 ± 4.20	15.16 ± 4.25	<0.001
Complications, n(%)				
UGB	10 (13.89)	50 (15.15)	2 (3.64)	0.069
SBP*	1 (1.39)	5 (1.52)	0 (0.00)	1.000
HE	0 (0.00)	12 (3.64)	0 (0.00)	0.093

Abbreviations: SBP, systolic blood pressure; DBP, diastolic blood pressure; MELD, model for end stage liver disease; UGB, upper gastrointestinal bleeding; SBP*, spontaneous bacterial peritonitis; HE, hepatic encephalopathy; INR, international normalized ratio; eGFR, estimated GFR; ALB, albumin; AST, aspartate aminotransferase; ALT, alanine aminotransferase; GGT, gamma-glutamyl transferase; ALP, alkaline phosphatase.

### Association Between Macrocytic Anemia and MELD Score

In univariate regression analysis, we found that a significant correlation was present both macrocytic anemia and the MELD score (*β* = 2.20, 95% CI: 0.99–3.41, *p* < 0.001), normocytic group was used as a normalization control (Table 2). Moreover, this association persisted (*β* = 2.31, CI:1.09–3.52, *p* < 0.001) after adjustment for sex and age in Model I (*β* = 2.40, CI:1.06–3.74, *p* < 0.001) after adjustment for sex, age, smoking, drinking, SBP, DBP in Model II in multivariate analysis (Table 3).

**TABLE 2:** Univariate analysis for MELD score.

	Statistics	β (95%CI)	*p*-value
Sex			
Female	118 (25.82%)	Ref	
Male	339 (74.18%)	−0.57 (−1.58, 0.43)	0.2627
Age	65.5 ± 12.9	−0.0 (−0.0, 0.0)	0.786
Smoking	149 (32.6%)	0.5 (−0.5, 1.4)	0.315
Drinking	132 (28.9%)	0.5 (−0.5, 1.4)	0.354
Diabetes	77 (16.8%)	−0.7 (−1.8, 0.5)	0.271
Hypertension	76 (16.6%)	−0.8 (−2.0, 0.4)	0.179
Hemoglobin, g/L			
>90	311 (68.1%)	Ref	
60–90	125 (27.4%)	0.6 (−0.4, 1.6)	0.248
<60	21 (4.6%)	1.9 (−0.2, 4.0)	0.074
Blood glucose	6.1 ± 3.0	−0.0 (−0.2, 0.1)	0.827
SBP	128.6 ± 19.1	−0.0 (−0.0, 0.0)	0.520
DBP	77.6 ± 11.8	0.0 (−0.0, 0.0)	0.988
MCV, fl	91.20 ± 9.86	0.04 (0.00, 0.09)	0.0485
Anemia classification			
Normocytic anemia	330 (72.21%)	Ref	
Macrocytic anemia	72 (15.75%)	2.20 (0.99, 3.41)	0.0004
Microcytic anemia	55 (12.04%)	0.34 (−1.01, 1.70)	0.6195

Abbreviations: MELD, model for end stage liver disease; *β* estimated coefficient; 95% CI 95% confidence interval; SBP, systolic blood pressure; DBP, diastolic blood pressure; MCV, mean corpuscular volume; fl.

**TABLE 3:** Relationship between MCV and MELD in different models.

Variable	Crude model		Model I		Model II	
	β (95%CI)	*p*-value	β (95%CI)	*p*-value	β (95%CI)	*p*-value
MCV, fl	0.04 (0.00, 0.09)	0.0485	0.05 (0.00, 0.09)	0.0428	0.05 (0.00, 0.10)	0.0367
Anemia classification						
Normocytic anemia	References		References		References	
Macrocytic anemia	2.20 (0.99, 3.41)	<0.001	2.31 (1.09, 3.52)	<0.001	2.40 (1.06, 3.74)	<0.001
Microcytic anemia	0.34 (−1.01, 1.70)	0.6195	0.39 (−0.97, 1.74)	0.5762	0.29 (−1.17, 1.76)	0.6958

Abbreviations: CI, confidence interval.

Model I adjusted for Sex and Age. Model II adjusted for Sex, Age, Smoking, Drinking, SBP, DBP.

### Association Between MCV and MELD Score

**FIGURE 2:**
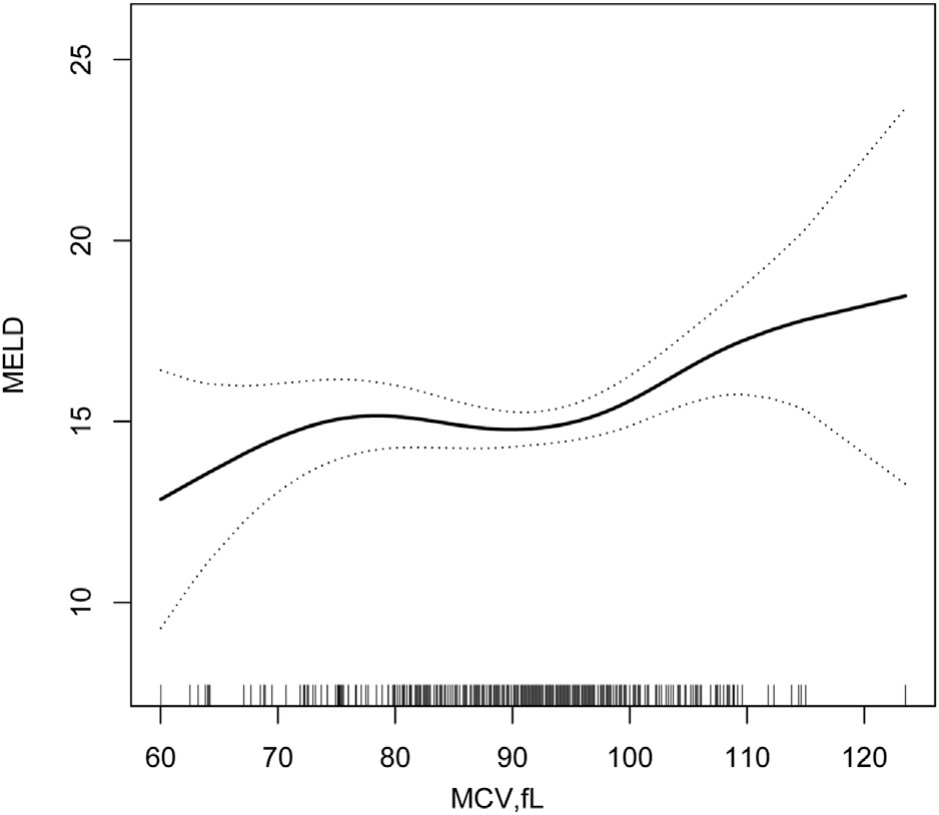
Two-piece piecewise regression and smooth curve-fitting for association between MCV and MELD stratified.

The two-piece wise smooth curve for MCV-MELD association in decompensated HBV associated cirrhosis. MCV negatively correlated with MELD when the MCV was smaller or equal than 98.2 fl (regression coefficient = 0.793,95% CI—0.1, 0.1). There was a strong positive correlation if MCV greater than 98.2 fl (regression coefficient = 0.008,95% CI 0.1, 0.4) (Figure 2; Table 4).

**TABLE 4:** Threshold Effect Analysis of MCV and MELD using Piece-wise Linear Regression.

Inflection point of MCV	Effect size(*β*)	95%CI	*p* Value
<98.2	0.0	−0.1 to 0.1	0.793
≥98.2	0.2	0.1 to 0.4	0.008

Effect: MELD Cause: MCV

adjusted :Sex, Age, Smoking, Drinking, SBP, DBP.

## Discussion

In this study the analysis was done retrospectively, we suggested that macrocytic anemia (MCV >100 fl) related to the degree of liver damage in decompensated HBV associated cirrhosis patients. This variability persists even after adjusting for age, gender, smoking, drinking, SBP and DBP.

Positive association between MCV and MELD was found among HBV-associated decompensated cirrhosis. Our findings by the two-piece piece-wise regression model to display the relationship between MCV and MELD as non-linear relationship. Positive correlation was observed when the MCV was higher than 98.2 fl, while negative correlation occurred when the MCV was lower than 98.2 fl.

Macrocytosis, which is also known as MCV >100 fl, is not necessarily corelated with anemia. Moreover, in most of the cases it is unattached to anemia (Eisenga et al., 2019). We select anemia as an inclusion criteria in our model because 70% patients in our study have anemia. In patients with advanced chronic liver disease, a research reported that 66% of the population suffers from anemia of different etiologies, this result was consistent with our study (Scheiner et al., 2020). Furthermore, the cause of occurred anemia in patients with liver cirrhosis due to shortened erythrocyte survival, a lack of hematopoietic cytokine, gastrointestinal bleeding, bone marrow disorders. All these suggest serious impairment of liver function and a high risk of death.

The significance of macrocytosis remains an underestimated issue in the past. Only a small number of studies had relevant reports (Bourlon et al., 2016; Kloth et al., 2016; Lee et al., 2020). An article indicated that a high MCV was associated with increased risk of death from liver cancer in males (Yoon et al., 2016). One study with small sample size also found that the MCV was notably higher in chronic hepatic failure patients than in healthy individuals (Remková and Remko, 2009). A precious published finding also showed that macrocytic anemia was related to the degree of liver damage in patients with decompensated HBV associated cirrhosis (Yang et al., 2018). These results fit in with our study.

Macrocytosis is considered a structural and functional abnormality of the erythrocyte membrane. Several potential pathological mechanisms may explain our observations. First, irrespective of the etiology vitamin deficits is common in patients with cirrhosis, such as vitamin B12 and folate deficiency (Gupta et al., 2019; Ohfuji et al., 2021), macrocytic anemia usually occurs due to liver dysfunction, low intakes of dietary, low uptake and increased catabolism. Vitamin B12 and folate coenzymes deficiency are known to cause delayed in DNA synthesis and eventually results in macrocytic anemia (Green and Dwyre, 2015; Lanier et al., 2018). Second, oxidative stress has been identified as an pivotal pathophysiological mechanism in chronic viral hepatitis B (Uchida et al., 2020). Because red blood cell is thought to be tightly related to whole-body antioxidant capacity (Tsantes et al., 2006). Oxidative stress decreases the RBC capacity to deform, reduces blood flow in microcirculation and compromises oxygen supply to certain tissues (Mohanty et al., 2014; Skjelbakken et al., 2014). Moreover, There are various factors that affect erythrocyte morphology in liver disease, such as etiology, severity of hepatic impairment, and use of drugs. There are many complicated mechanisms that affect the shape of red blood cells. These mechanisms may allow to perform effectively their independent or collaborative functions. Nevertheless, it’s clear that macrocytic anemia has a positive correlation with the degree of liver damage in patients with decompensated HBV associated cirrhosis.

The MELD score is approved for assessing the degree of liver diseases. These variables include prothrombin time, INR, serum bilirubin and creatinine level. MELD score changes with variations in these variables. Higher MELD scores associate with increased risks of death and hepatic events in cirrhosis. In our study, among the parameters of MELD score, bilirubin and INR showed an increase on patients with macrocytic anemia. However, there was no remarkable difference with creatinine and eGFR. Therefore, macrocytic anemia may not be relevant to renal injury in patients with decompensated HBV associated cirrhosis.

Several study limitations are noted. First, the main limitation of this study lies in its retrospective observational nature, the cross-sectional nature of our study does not permit the determination of causality between MCV and MELD. Second, this study included only Chinese participants, and therefore these findings may not be generalizable to other biogeographic ethnic groups. Third, we did not perform an analysis on the data of folate, serum vitamin B12 and reticulocyte count, which could provide a better understanding of macrocytic anemia in cirrhotic patients.

## Conclusion

Macrocytic anemia was highly correlated with the degree of hepatic dysfunction and may be a reliable predictor for mortality in patients with decompensated HBV associated cirrhosis. We found a non-linear relationship between MCV and MELD. Moreover, further large-scale, well-designed and multicenter studies need to be conducted to confirm our conclusions, it is important to evaluate and investigate this association and to gain insight the underlying mechanisms.

## Data Availability

The raw data supporting the conclusion of this article will be made available by the authors, without undue reservation.
